# Bronchial artery embolization followed by bronchoscopic electrosurgical snare resection for diagnosis and treatment of primary pulmonary osteosarcoma: case report and literature review

**DOI:** 10.3389/fmed.2026.1931048

**Published:** 2026-09-25

**Authors:** Jiali Zhang, Lu Wang, Gang Yin, Huashun Li, Jiangqin Song, Fuying Hu

**Affiliations:** 1 Department of Pulmonary and Critical Care Medicine, Tianmen Hospital Affiliated to Wuhan University of Science and Technology, Tianmen, Hubei, China; 2 Department of Pulmonary and Critical Care Medicine, Tianmen First People's Hospital, Tianmen, Hubei, China; 3 Department of Trauma Surgery, Tianmen First People's Hospital (Tianmen Hospital Affiliated to Wuhan University of Science and Technology), Tianmen, Hubei, China; 4 Department of Pathology, Tianmen First People's Hospital (Tianmen Hospital Affiliated to Wuhan University of Science and Technology), Tianmen, Hubei, China; 5 Hubei Key Laboratory of Occupational Hazard Identification and Control, Wuhan University of Science and Technology, Wuhan, Hubei, China; 6 Department of Laboratory Medicine, Tianmen First People's Hospital (Tianmen Hospital Affiliated to Wuhan University of Science and Technology), Tianmen, Hubei, China; 7 Department of Pulmonary and Critical Care Medicine, Tianmen First People's Hospital (Tianmen Hospital Affiliated to Wuhan University of Science and Technology), Tianmen, Hubei, China

**Keywords:** airway obstruction, bronchial artery embolization, bronchoscopy, case report, electrosurgical snare resection, extraskeletal osteosarcoma, primary pulmonary osteosarcoma

## Abstract

Primary pulmonary osteosarcoma (PPOS) is an extremely rare extraskeletal osteosarcoma. Because its clinical and radiological features are nonspecific, establishing a diagnosis is challenging. We report the case of a 58-year-old man who presented with a 1-month history of cough and 5 days of dyspnea. Initial chest computed tomography showed destructive changes in the left lung and an endobronchial lesion in the left main bronchus. To relieve airway obstruction and obtain diagnostic tissue, a sequential strategy of bronchial artery embolization followed by bronchoscopic electrosurgical snare resection was used. Pathological examination showed a high-grade sarcoma with characteristic malignant osteoid matrix, chondroid differentiation, and numerous multinucleated tumor giant cells. Immunohistochemistry showed positivity for SATB2 and vimentin, with a Ki-67 proliferation index of approximately 30%. Whole-body bone scintigraphy, brain magnetic resonance imaging, and contrast-enhanced abdominal and pelvic computed tomography excluded a primary bone tumor and extrapulmonary metastasis, supporting the diagnosis of PPOS. After the procedure, dyspnea improved, follow-up chest computed tomography showed partial re-expansion of the left lung and disappearance of the left main bronchial lesion, and the patient was referred to a higher-level hospital for radiotherapy. He remained alive at 9-month follow-up after diagnosis. This case suggests that bronchial artery embolization-assisted bronchoscopic electrosurgical snare resection may provide a minimally invasive option for obtaining diagnostic tissue and relieving airway obstruction in selected hypervascular endobronchial tumors.

## 1 Introduction

Primary pulmonary osteosarcoma (PPOS) is an extremely rare malignant lung tumor and has been estimated to account for approximately 0.01% of all primary pulmonary tumors (1). Because characteristic clinical manifestations and imaging features are lacking, preoperative diagnosis is difficult, and pathological examination remains decisive for diagnosis (2). In previously reported cases, adequate tissue for diagnosis was often obtained surgically, which is invasive; obtaining sufficient diagnostic tissue through a minimally invasive approach remains clinically challenging. Here, we report a patient with PPOS in whom bronchial artery embolization (BAE) before bronchoscopic electrosurgical snare resection relieved airway obstruction and yielded adequate biopsy tissue. A narrative review of previously reported cases was also performed (Supplementary Table 1).

## 2 Case description

A 58-year-old man presented to the outpatient department of our hospital on December 15, 2025, with a 1-month history of cough and 5 days of dyspnea. Non-contrast chest computed tomography (CT) showed destructive changes in the left lung and a strip-like high-density space-occupying lesion within the left main bronchus (Figure 1A), and he was admitted with a suspected pulmonary space-occupying lesion. The patient reported dry cough without an obvious trigger 1 month earlier, with occasional small amounts of white mucoid sputum and intermittent blood-streaked sputum. Five days before presentation, he developed exertional dyspnea that was partially relieved by rest. He denied fever, night sweats, weight loss, and other discomfort. He had a history of hypertension with stable blood pressure control and denied chronic obstructive pulmonary disease, diabetes mellitus, coronary heart disease, previous malignancy, radiotherapy, or chemotherapy. He had smoked 20 cigarettes per day for 40 years. On admission, the respiratory rate was 25 breaths/min, and peripheral oxygen saturation was 77.0% on room air, without use of accessory respiratory muscles. Arterial blood gas analysis during nasal cannula oxygen therapy showed a pH of 7.426, a PaCO_2_ of 35.0 mmHg, a PaO_2_ of 81.2 mmHg, an oxygen saturation of 97.2%, and a lactate level of 1.2 mmol/L, indicating hypoxemia without carbon dioxide retention. Breath sounds were decreased bilaterally, particularly on the left side, and the remainder of the physical examination was unremarkable. Laboratory tests showed a high-sensitivity C-reactive protein level of 54.76 mg/L, erythrocyte sedimentation rate of 45 mm/h, interleukin-6 level of 173.65 pg/mL, carcinoembryonic antigen level of 6.20 ng/mL, and carbohydrate antigen 125 level of 35.70 U/mL.

**Figure 1:**
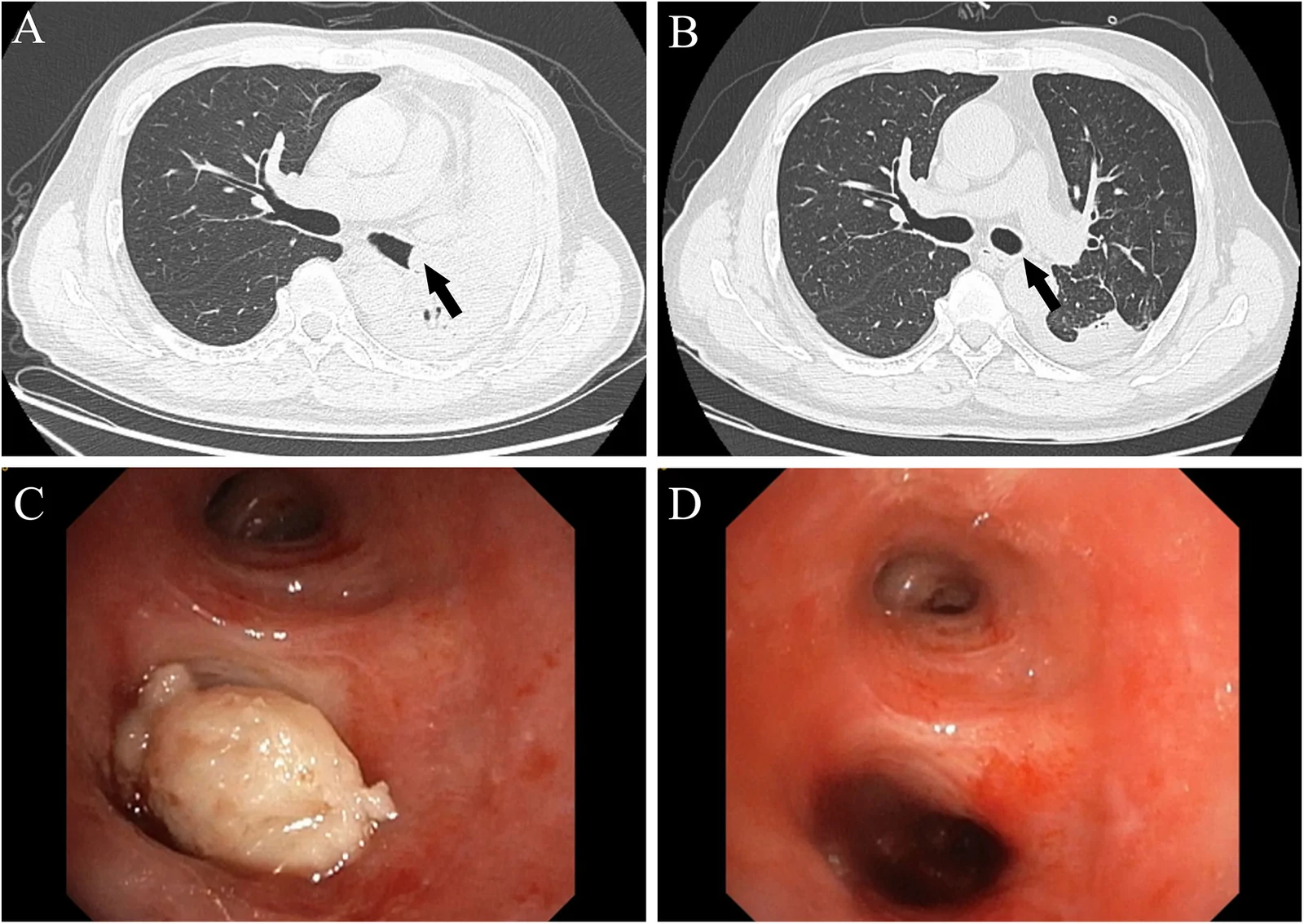
Chest CT and bronchoscopic findings before and after bronchial artery embolization (BAE) followed by bronchoscopic electrosurgical snare resection. Preoperative non-contrast chest CT at presentation (December 15, 2025) showing destructive changes with near-complete opacification of the left lung and a strip-like high-density shadow within the left main bronchial lumen (arrow) **(A)**; non-contrast chest CT obtained 1 day after endoscopic resection (December 20, 2025) showing partial re-expansion of the left lung and disappearance of the high-density shadow in the left main bronchus (arrow) **(B)**; preoperative bronchoscopy (December 17, 2025) showing a polypoid neoplasm covered with necrotic material in the distal left main bronchus, at the openings of the left upper and lower lobe bronchi **(C)**; postoperative bronchoscopy (December 22, 2025) showing a patent airway at the openings of the left upper and lower lobe bronchi, with inflammatory repair changes at the operative wound **(D)**.

### 2.1 Diagnostic assessment

Contrast-enhanced chest CT on December 16, 2025, showed a strip-like slightly hyperdense shadow in the left main bronchus with heterogeneous enhancement, distal bronchial occlusion, consolidation of most of the left lung, and left pleural effusion. Flexible bronchoscopy on December 17 showed an elevated lesion in the left main bronchial lumen, covered with necrotic material and prone to bleeding on contact. The patient's dyspnea did not improve. After multidisciplinary consultation with thoracic surgery, oncology, radiology, and anesthesiology, airway obstruction caused by a bronchial tumor was considered, and interventional bronchoscopic treatment was deemed indicated (3). Because the lesion appeared hypervascular, a sequential strategy of BAE followed by bronchoscopic electrosurgical snare resection was selected. The patient underwent BAE on December 18, 2025, and bronchoscopic electrosurgical snare resection on December 19, which relieved airway obstruction and provided sufficient tissue. Pathological examination of the resected specimen showed a high-grade sarcoma with characteristic tumor osteoid matrix (Figure 2). Immunohistochemistry showed SATB2 (+), vimentin (+), P16 (diffuse strong +), P53 (diffuse strong +), and a Ki-67 proliferation index of approximately 30%; S-100, SOX-10, MyoD1, MDM2, CD34, CD68, and SMA were negative (Figure 3).

**Figure 2:**
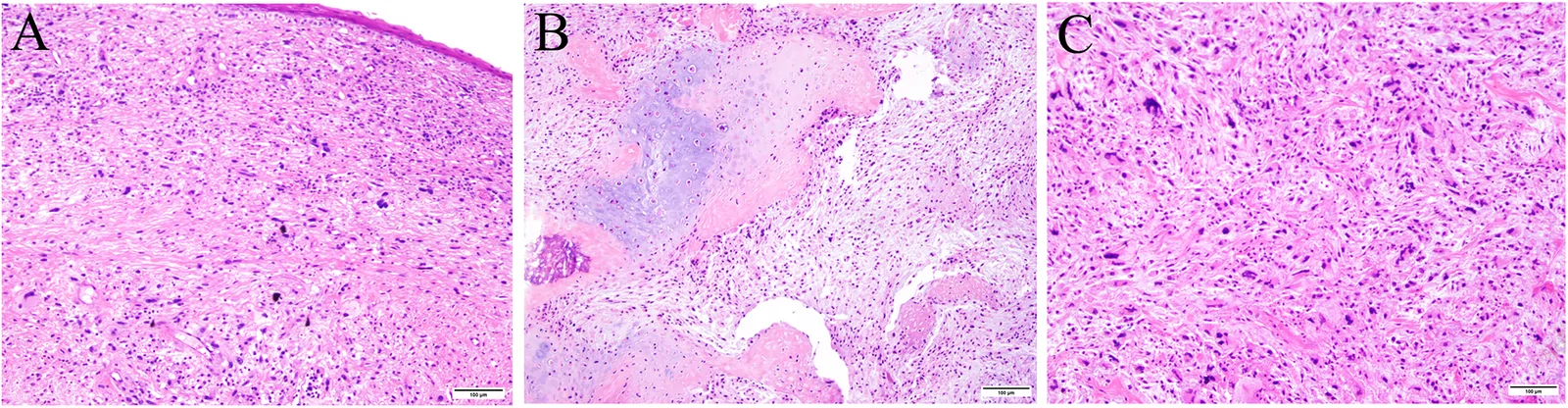
Histopathological examination (hematoxylin and eosin staining, × 100; scale bar = 100 *μ*m). The tumor surface was covered by normal squamous epithelium, and diffuse oval and spindle tumor cells were observed beneath the epithelium with a small amount of inflammatory cell infiltration in the stroma **(A)** Tumor osteoid matrix and chondroid differentiation were present, showing multidirectional mesenchymal differentiation **(B)** Tumor cells showed marked atypia with numerous multinucleated tumor giant cells **(C)**.

**Figure 3:**
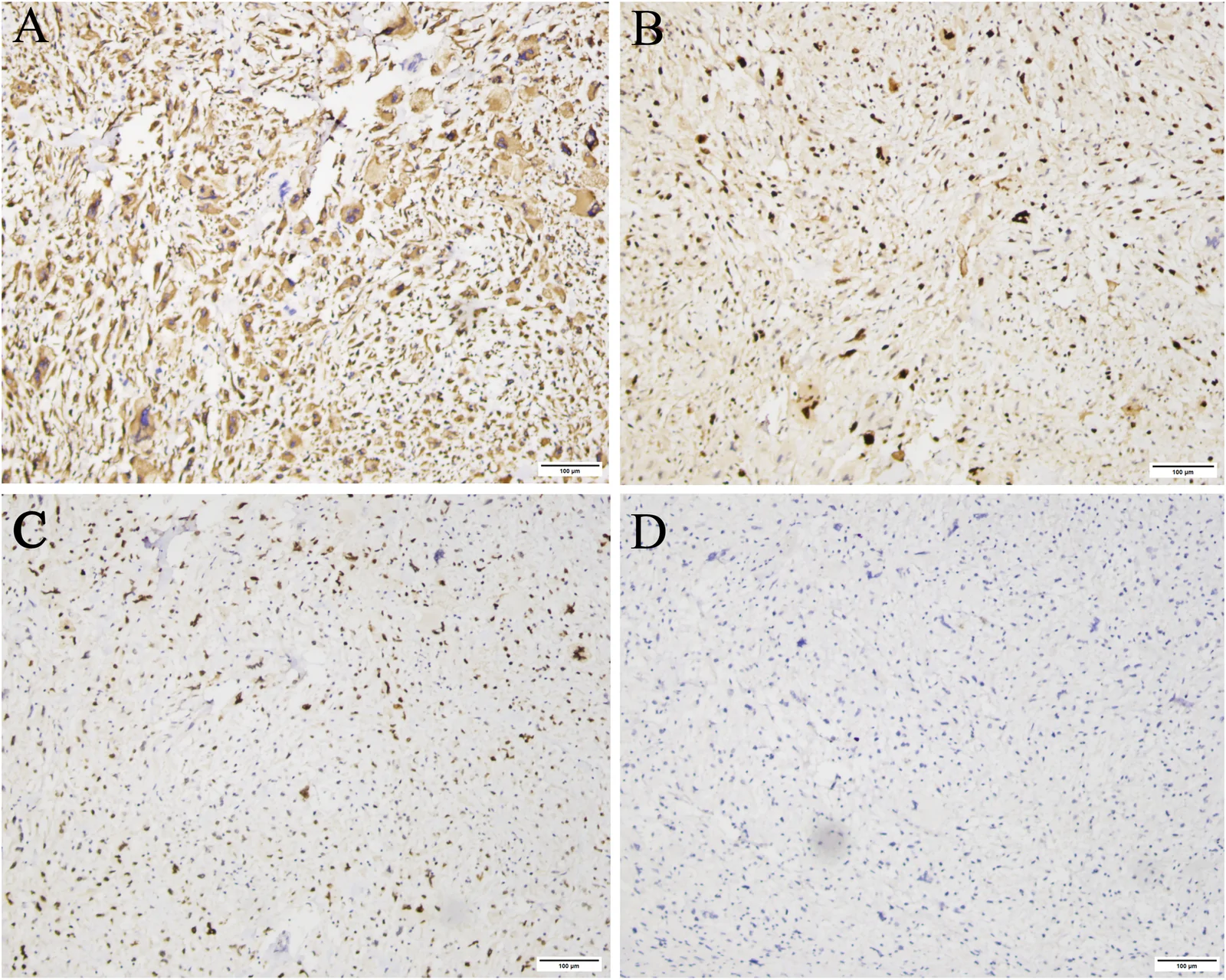
Immunohistochemical examination (× 100; scale bar = 100 *μ*m). Tumor cells showed cytoplasmic positivity for vimentin **(A)**, nuclear positivity for SATB2 **(B)**, a Ki-67 proliferation index of approximately 30% **(C)**, and negativity for MyoD1 **(D)**.

To exclude a primary bone tumor and extrapulmonary metastasis, contrast-enhanced abdominal and pelvic CT on December 21, 2025, showed multiple renal calculi and cysts, a paracolic diverticulum near the ascending colon, and an appendicolith, without abnormalities in the liver, gallbladder, pancreas, spleen, bladder, prostate, or retroperitoneum. Brain magnetic resonance imaging on December 22 showed mild demyelinating changes in the cerebral white matter and inflammatory changes in the paranasal sinuses, with no abnormal enhancement in the brain parenchyma or meninges. Whole-body bone scintigraphy with ^99m^Tc-MDP SPECT/CT on December 23 showed increased uptake in the right wrist joint, sternum, and L5 vertebra, which was considered degenerative; no abnormal radioactive concentration was detected in the remaining skeleton. Given the bronchial origin of the lesion, the absence of systemic involvement on imaging, and the typical osteosarcoma morphology and immunophenotype, the final diagnosis was PPOS.

### 2.2 Therapeutic intervention

After admission, the patient received high-flow oxygen through a reservoir mask at 15 L/min, bronchodilator therapy, anti-infective treatment, expectorant therapy, and other supportive measures. Because dyspnea persisted despite supportive treatment, multidisciplinary discussion concluded that interventional bronchoscopic treatment was indicated. Because of the rich vascular supply of the lesion, BAE was performed before bronchoscopic electrosurgical snare resection. At 9:00 a.m. on December 18, 2025, BAE was performed under local anesthesia through right femoral artery puncture. A COOK cobra catheter and a CORDIS pig catheter were introduced, and angiography of the aortic arch, bilateral bronchial arteries, and left subclavian artery was performed. Angiography showed two left bronchial arteries: one was thin, shared a common trunk with the right bronchial artery, and originated from an intercostal artery; the other arose separately, showed irregular staining in the late parenchymal phase, and had a peripheral bronchial artery-pulmonary artery fistula. A Terumo microcatheter (MC-PE27131) was advanced superselectively into the distal segments of the two responsible vessels, which were embolized with 350 *μ*m polyvinyl alcohol particles. Post-embolization angiography showed disappearance of the irregular staining and the arterial fistula. The puncture site was compressed and dressed, and the right lower limb was immobilized for 8 h.

Thirty hours later, bronchoscopic electrosurgical snare resection was performed under general anesthesia with a laryngeal mask airway. The glottis opened and closed well, and the trachea was patent. Lidocaine was instilled segmentally into the bronchial tree to reduce coughing. A neoplasm completely obstructing the distal left main bronchus was observed. The surface of the lesion was covered with purulent secretions and appeared richly vascularized. Tumor tissue was cut at multiple points with an electrosurgical needle knife without obvious bleeding, followed by repeated electrosurgical snare resection and cryoextraction. After stepwise endoscopic tumor removal, the fragments were retrieved with a basket. Grossly complete tumor clearance was achieved, and the left upper and lower lobe bronchi became patent. The tumor base was located on the bronchial wall at the opening of the basal segment of the left lower lobe, where repeated cryotherapy was applied for 40 s each time. After airway patency was restored, abundant purulent secretions drained from the left upper and lower lobe bronchi and were repeatedly lavaged with normal saline and aspirated. Only minor intraoperative bleeding occurred and stopped after local administration of epinephrine and iced saline. After 30 min of postoperative observation, vital signs remained stable, the laryngeal mask was removed, and the patient returned to the ward. Postoperatively, intravenous dexamethasone and furosemide were administered to reduce bronchial mucosal edema, and nebulized budesonide plus acetylcysteine was used for local anti-inflammatory treatment and sputum dilution. After the pathological diagnosis was established at our hospital, the patient was transferred to a higher-level hospital for further oncologic evaluation and treatment on December 28, 2025, and subsequently received radiotherapy.

### 2.3 Follow-up and outcomes

After the procedure, dyspnea improved markedly, and peripheral oxygen saturation remained between 96% and 99%. Follow-up chest CT on December 20, 2025, showed partial re-expansion of the left lung and disappearance of the strip-like high-density shadow in the left bronchus. Repeat bronchoscopy on December 22 showed a patent left main bronchial lumen and inflammatory repair changes at the operative wound. Pre- and postoperative imaging and bronchoscopic findings are compared in Figure 1. After diagnosis, the patient was referred to a higher-level hospital for radiotherapy. At the most recent follow-up on August 26, 2026, the patient remained alive 9 months after diagnosis. The complete clinical, diagnostic, and therapeutic timeline is shown in Figure 4.

**Figure 4:**
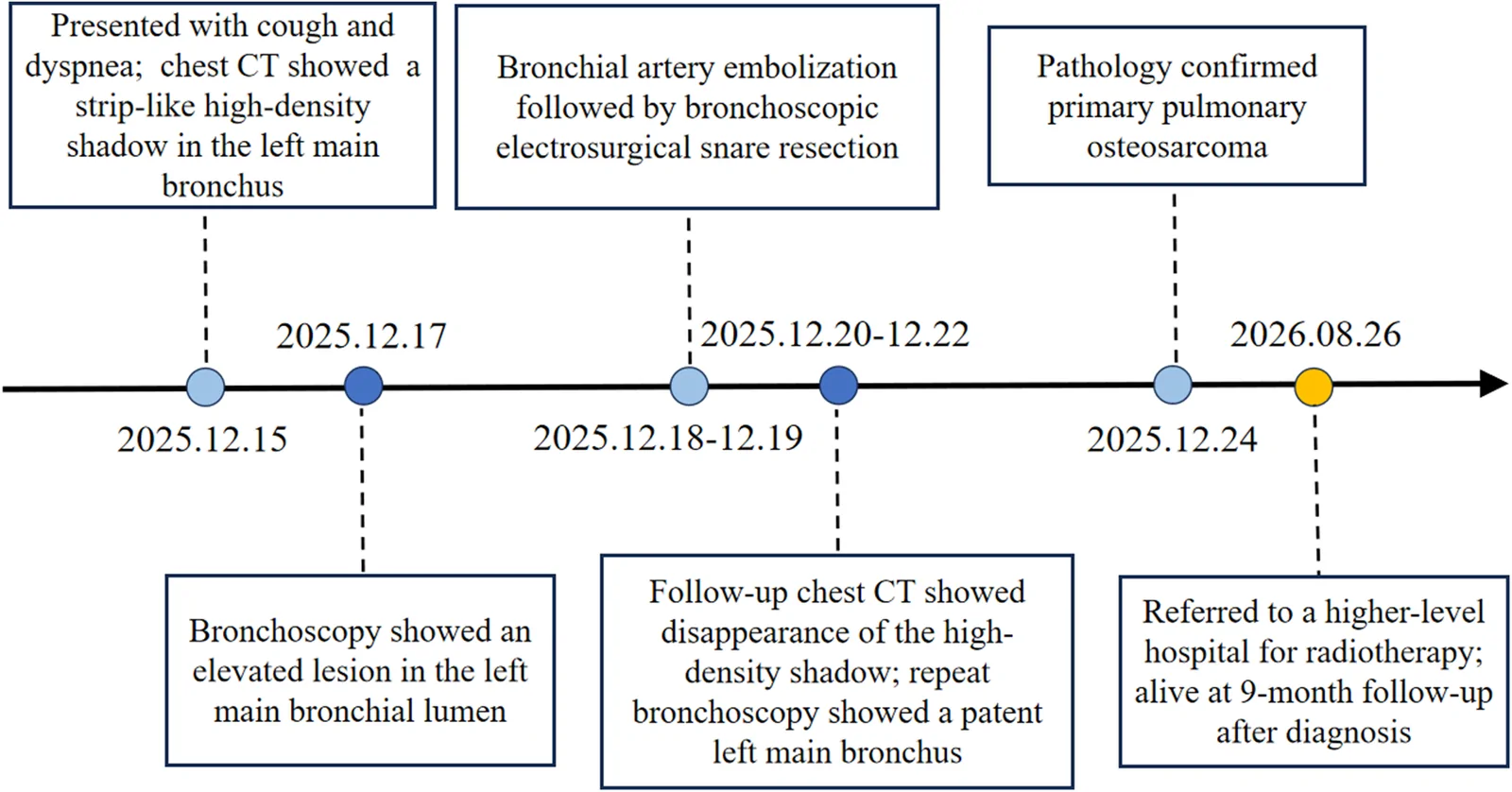
Clinical, diagnostic, and therapeutic timeline of the patient with primary pulmonary osteosarcoma.

## 3 Discussion

PPOS is a highly malignant soft tissue tumor and the rarest histological subtype among primary pulmonary sarcomas (2). In most previously reported cases, surgical specimens were required for diagnosis. In the present case, BAE followed by bronchoscopic electrosurgical snare resection successfully removed tumor tissue, restored airway patency, and provided sufficient tissue for pathological diagnosis. This sequential approach therefore served both diagnostic and therapeutic purposes. Similar strategies of preoperative BAE followed by bronchoscopic resection have been reported for hypervascular endobronchial tumors (4), and the present case extends the application of this established interventional approach to PPOS, an extremely rare tumor.

A systematic literature search of PubMed, China National Knowledge Infrastructure (CNKI), and the Chinese Medical Journal Network was performed from database inception to June 28, 2026, using the exact phrases “pulmonary osteosarcoma”, “lung osteosarcoma”, “extraskeletal osteosarcoma of the lung”, and “primary pulmonary osteosarcoma” (or the corresponding Chinese terms), supplemented by citation searching of reference lists. Eligible reports described pathologically confirmed osteosarcoma arising primarily in the lung, with skeletal primary tumors excluded and original case-level data available, published in English or Chinese. The database search yielded 217 records; after deduplication and screening, 12 articles were included, and citation searching identified 19 additional eligible reports. In total, 31 articles reporting 40 cases of PPOS were included (Supplementary Table 1; the selection process is shown in the PRISMA flow diagram, Supplementary Figure 3). Among the 40 previously reported cases, 28 were male and 12 were female (male-to-female ratio, 2.3:1), with a median age of 61 years (range, 17–83 years). The most common presenting symptoms were chest pain (*n* = 13), cough (*n* = 12), and hemoptysis (*n* = 8); six patients were asymptomatic and detected incidentally. Tumors arose more frequently in the left lung (*n* = 26) than in the right lung (*n* = 13), with a median maximum diameter of 7.0 cm (range, 1.8–25.0 cm). The diagnosis was most often established by surgical resection (*n* = 24) or at autopsy (*n* = 6). The pathogenesis of PPOS remains unclear. Existing reports suggest that local trauma or radiotherapy may be potential risk factors, although the level of evidence is limited (5); the present patient had no history of trauma, chemotherapy, or radiotherapy. Because the clinical manifestations of PPOS lack specificity, imaging is important for screening and localization, but pathological examination remains essential for diagnosis. Chest CT is the main imaging modality for PPOS. Lesions often appear as large lobulated pulmonary soft-tissue masses; calcification on CT was documented in 11 of the 15 cases with available CT descriptions, while adjacent bony structures are usually intact without destruction. Hemorrhage and necrosis may occur within the mass, and contrast enhancement is often heterogeneous (6).

Grossly, PPOS is often a gray-white solid mass with a firm consistency and is usually large; reported diameters range from 1.8 to 25 cm, with a median size of approximately 6 cm (1). Microscopically, malignant primitive spindle cells and osteoid matrix are typical pathological hallmarks (7). Immunohistochemistry is important for diagnosis and differential diagnosis. Vimentin is a marker of mesenchymal tumors (8) and is frequently positive in PPOS; the tumor in this case was vimentin-positive, consistent with mesenchymal origin SATB2, a sensitive marker of osteoblastic differentiation, is a nuclear matrix-associated transcription factor involved in osteoblast lineage differentiation. SATB2 is highly expressed in extraskeletal osteosarcoma, and Conner and Hornick reported a positivity rate of 89% (9). SATB2 positivity in this case provided strong immunohistochemical support for the diagnosis. However, SATB2 is not absolutely specific for osteosarcoma; some myogenic and fibroblastic soft tissue sarcomas, fibro-osseous lesions, and colorectal adenocarcinomas may also show positivity (10). Therefore, its diagnostic value must be interpreted together with morphology and other immunohistochemical markers. In the present case, negativity for S-100 and SOX-10 did not support metastatic melanoma, MyoD1 negativity did not support rhabdomyosarcoma, MDM2 negativity did not support dedifferentiated liposarcoma, CD68 negativity did not support histiocytic tumors, and SMA negativity did not support smooth muscle tumors (11).

When imaging, pathology, and immunohistochemistry are compatible with PPOS, a primary pulmonary origin should be considered, but systemic evaluation is required to exclude pulmonary metastasis from skeletal osteosarcoma or another primary tumor. Whole-body bone scintigraphy is useful for detecting occult skeletal lesions, and brain magnetic resonance imaging and abdominal and pelvic CT may be used to evaluate specific sites. PET/CT can be considered when further assessment of systemic tumor involvement is needed. If no primary bone lesion or other primary tumor is detected and there is no clinical evidence of secondary tumor involvement, a diagnosis of PPOS may be considered. Currently, diagnosis of PPOS requires the following criteria: (1) the tumor shows a pure osteosarcomatous histological pattern; (2) the tumor produces osteoid or bone matrix; and (3) the tumor originates in the lung, with metastasis from skeletal osteosarcoma excluded (2).

No standardized diagnostic or therapeutic guidelines for PPOS are currently available. Reported treatments are mainly surgical resection, with chemotherapy or radiotherapy used in some patients. In the present case, bronchoscopy showed that a neoplasm in the left main bronchus completely obstructed the lumen, meeting the diagnostic criteria for central airway obstruction (CAO) (12). For symptomatic CAO, the 2024 American College of Chest Physicians guideline recommends therapeutic bronchoscopy as an important modality for symptom improvement (3). This patient had significant dyspnea and met indications for interventional treatment. However, endoscopic resection of hypervascular endobronchial tumors carries a risk of severe bleeding. In the present case, both radiologic and bronchoscopic findings suggested marked tumor vascularity: contrast-enhanced chest CT showed heterogeneous enhancement of the endobronchial lesion, indicating vascularized viable tumor tissue, and bronchoscopy revealed a lesion covered with necrotic material that bled easily on contact. BAE is an effective minimally invasive method for controlling bleeding, and preoperative embolization of tumor-feeding arteries can reduce the risk of intraoperative hemorrhage. A retrospective study of 24 patients with CAO reported no severe bleeding during endoscopic tumor resection after BAE pretreatment (4). In the present case, BAE was performed first to reduce tumor blood supply and decrease bleeding risk; angiography confirmed the hypervascular nature of the tumor, demonstrating irregular tumor staining in the late parenchymal phase and a peripheral bronchial artery–pulmonary artery fistula. Bronchoscopic electrosurgical snare resection was then performed, which provided sufficient tumor tissue, relieved airway obstruction, and led to the pathological diagnosis of PPOS, with only minor intraoperative bleeding. Based on our experience, pre-bronchoscopic BAE may be considered for patients with central airway tumors in whom contrast-enhanced CT or bronchoscopic findings suggest marked tumor vascularity and in whom a high risk of procedure-related bleeding is anticipated. Regarding subsequent oncologic management, surgical resection was discussed at the multidisciplinary consultation. Because bronchoscopic debulking achieved grossly complete removal of the endobronchial tumor with restoration of airway patency, the patient was referred to a higher-level hospital for further evaluation, where additional surgical resection was considered unnecessary and radiotherapy was selected as the definitive local treatment. This strategy achieved both airway palliation and diagnostic tissue acquisition, highlighting the potential value of interventional pulmonology in rare and diagnostically challenging pulmonary tumors.

The prognosis of PPOS is difficult to predict because of its rarity, but overall outcomes appear poor. Among the 40 cases identified in our literature review (Supplementary Table 1), 19 patients died within 1 year after symptom onset or diagnosis, suggesting rapid progression and poor prognosis. Prognosis may be associated with maximum tumor diameter, the presence or absence of calcification, and the extent of surgical resection (13, 14). Huang et al. reported that a tumor diameter greater than 7 cm and absence of osteoid tissue microscopically were associated with poor prognosis (1). Long-term survival has also been reported in isolated cases; Wen et al. described a 17-year-old male patient with PPOS who remained alive 8 years after surgery followed by chemotherapy, suggesting that younger age and timely intervention may improve outcomes in selected patients (2).

Several limitations of this report should be noted. First, this is a single-case report, and the favourable outcome observed herein may not be reproducible in all patients. The sequential strategy was adopted in the present case given the central location and hypervascular features of the tumour demonstrated on contrast-enhanced CT and bronchoscopy. For patients with less vascular or peripherally located tumours, or in centres without ready access to interventional radiology and therapeutic bronchoscopy, the risk-benefit balance of this approach may differ. Clinical management should be tailored to individual patients and local institutional expertise. Second, follow-up duration in this case is only 9 months; therefore, no conclusions regarding long-term prognosis can be drawn, and the patient remains under active follow-up.

## 4 Conclusion

PPOS is a rare and highly aggressive pulmonary malignancy with an overall poor prognosis. The present patient developed cough and dyspnea, and chest CT showed a high-density lesion in the left main bronchus with destructive changes in the left lung. BAE followed by bronchoscopic electrosurgical snare resection relieved dyspnea, eliminated the intrabronchial high-density lesion, and promoted partial re-expansion of the left lung. Pathological examination confirmed PPOS, and the patient subsequently received radiotherapy at a higher-level hospital and was alive at 9-month follow-up after diagnosis. This case suggests that, in selected patients with hypervascular endobronchial lesions, BAE followed by bronchoscopic electrosurgical snare resection may help relieve airway obstruction while obtaining diagnostic tissue.

## Patient perspective

I initially developed a cough but did not take it seriously. When dyspnea developed, I sought medical attention. I was alarmed upon learning of the bronchial mass and the severity of my airway obstruction. Interventional bronchoscopy was promptly performed, which markedly relieved my dyspnea. I was subsequently transferred to a higher-level hospital for radiotherapy. My condition has remained favorable to date.

## Data Availability

The original contributions presented in the study are included in the article/Supplementary Material, further inquiries can be directed to the corresponding authors.

## Supplementary material

Supplementary Figure 1 Bronchoscopic electrosurgical snare resection showing the snare encircling the tumor tissue **(A)**; the resected tumor tissue obtained by electrosurgical snare resection **(B)**; the tumor base located at the opening of the basal segment of the left lower lobe with a patent airway after resection **(C)**.

Supplementary Figure 2 Immunohistochemical examination (× 100; scale bar = 100µm). Tumor cells showed cytoplasmic positivity for vimentin **(A)**; nuclear positivity for SATB2 **(B)**; a Ki-67 proliferation index of approximately 30% **(C)**; MyoD1 negativity **(D)**; P16 diffuse strong positivity **(E)**; P53 diffuse strong positivity **(F)**; S-100 negativity **(G)**; SOX-10 negativity **(H)**; MDM2 negativity **(I)**; CD34 negativity **(J)**; CD68 negativity **(K)**; SMA negativity **(L)**.

Supplementary Figure 3 PRISMA 2020 flow diagram of the literature selection process. Records were identified through database searching (*n* = 217), including PubMed (*n* = 149), the Chinese Medical Journal Network (*n* = 47), and China National Knowledge Infrastructure (CNKI) (*n* = 21), supplemented by citation searching of reference lists (*n* = 23). After removal of duplicates (*n* = 4) and screening according to the predefined eligibility criteria, 31 articles reporting 40 cases of primary pulmonary osteosarcoma were included (Supplementary Table 1).
