# Supplementary material for: Bronchial artery embolization followed by bronchoscopic electrosurgical snare resection for diagnosis and treatment of primary pulmonary osteosarcoma: case report and literature review
Source: Front Med (Lausanne). 2026 Sep 25;13:1931048. doi: 10.3389/fmed.2026.1931048 (PMC13650938; doi:10.3389/fmed.2026.1931048)
Supplement: Supplementary file 1 [file Table1.docx]

**Supplementary Table 1. Reported cases of primary pulmonary osteosarcoma**

| **Author** | **Sex / Age** | **Chief complaints** | **Lesion site and size** | **Procedure to make definite diagnosis** | **Immunohistochemical findings** | **Calcification on CT scan** | **Bone scintigraphy uptake** | **Treatment** | **Prognosis** |
| --- | --- | --- | --- | --- | --- | --- | --- | --- | --- |
| Greenspan (1) | Female / 35 | Dyspnea, hemoptysis | Left main bronchus, 7.0 cm | Autopsy | ND | ND | ND | No radical treatment | Died 11 months after symptom onset |
| Yamashita et al. (2) | Female / 74 | Progressive asthmatic symptoms | Left lung replaced by a giant mass | Autopsy | ND | ND | ND | No radical treatment | Died 6 months after symptom onset |
| Nosanchuk and Weatherbee (3) | Male / 66 | Chest pain, hemoptysis | Most of the left upper and lower lobes involved | Autopsy | ND | ND | ND | No radical treatment | Died 4 months after symptom onset |
| Reingold and Amromin (4) | Female / 56 | Fever, chills, chest pain | Left upper lung, 7.0 × 5.0 × 4.0 cm | Surgery | ND | ND | ND | Surgery | Alive 14 weeks after surgery |
|  | Male / 62 | Fever | Right middle lobe, 6.0 × 6.0 × 4.0 cm | Autopsy | ND | ND | ND | Chemotherapy | Died 7 months after symptom onset |
| Nascimento (5) | Female / 77 | Asymptomatic | Right middle lobe, 4.0 cm | Surgery | ND | ND | ND | Surgery | Died 6 months after surgery |
|  | Male / 72 | Asymptomatic | Right middle lobe, 5.5 cm | Surgery | ND | ND | ND | Surgery | Died 10 months after surgery |
| Bagaric and Belicza (6) | Female / 49 | ND | Right lower lung | ND | ND | ND | ND | ND | Died |
| Saito et al. (7) | Male / 83 | Chest pain | Right middle lobe, 10.0 cm | ND | ND | ND | ND | ND | Died 4 months after symptom onset |
| Zhu et al. (8) | Male / 48 | Cough, chest pain | Right lung, 6.5 × 5.5 × 5.5 cm | Surgery | ND | ND | ND | Surgery | ND |
| Colby et al. (9) | Female / 77 | Pneumonia | Right middle lobe, 4.0 × 3.0 cm | ND | ND | ND | ND | ND | Died 6 months after surgery |
|  | Male / 51 | Cough | Left lower lobe, 11.0 × 7.0 cm | ND | ND | ND | ND | ND | Alive 6 months after surgery |
|  | Male / 61 | Respiratory symptoms | Right lung, 16.0 × 10.0 cm | ND | ND | ND | ND | ND | Died |
| Loose et al. (10) | Female / 45 | Left-sided chest pain | Left lower lung, 5.5 × 4.0 × 3.0 cm | Surgery | Vimentin (+), MSA(−), CK (−), S-100 (−), Desmin (−), EMA(−) | (−) | ND | Surgery | Alive 2 months after surgery |
|  | Male / 54 | Left subscapular back pain with paresthesia in the left axilla and triceps | Left upper lung, 10.0 × 9.0 × 4.5 cm | Surgery | Vimentin (+), MSA (+), CK (−), S-100 (−), Desmin (−), EMA (−) | (+) | Performed after surgery | Surgery, radiotherapy, and chemotherapy | Alive 7 months after surgery |
| Petersen (11) | Male / 70 | Asymptomatic | left lower lung, 5.0 × 3.5 × 6.0 cm | Surgery | ND | (+) | (+) | Surgery and radiotherapy | Alive 6 months after surgery |
| Stark et al. (12) | Male / 59 | Asymptomatic | Left lower lung, 6.5 × 7.0 × 11.0 cm | Surgery | ND | (+) | Performed after surgery | Surgery | ND |
| Bhalla et al. (13) | Male / 58 | Fever, cough | Left upper lung, 18.0 × 9.0 × 8.0 cm | Autopsy | ND | (+) | ND | No radical treatment | Died 1 month after diagnosis |
| Miller and Allen (14) | Male / 72 | ND | ND | Bronchoscopic biopsy | ND | ND | ND | Radiotherapy and chemotherapy | Died 12 months after symptom onset |
| Wei and Guo (15) | Male / 65 | Cough, hemoptysis | Left upper lung, 2.0 × 1.5 × 1.0 cm | Surgery | ND | ND | ND | Surgery | Alive 10 months after surgery |
| Wagner and Reyes (16) | Male / 48 | Chest pain | Right lower lobe | Surgery | Vimentin (+++), Keratins (−), EMA (−), Desmin (−), CEA (−) | (+) | ND | ND | Died 1 month after diagnosis |
| Sievert et al. (17) | Male / 56 | Numbness of the fingertips of the left hand | Left upper lung, 4.0 × 2.0 × 2.0 cm | Surgery | Vimentin (+), Actin (focal+), Desmin (focal+), CK (−), EMA (−), | (−) | Performed after surgery | Surgery | Alive 12 months after surgery |
| Chapman et al. (18) | Female / 33 | Cough, left-sided chest pain | Left lung, 5.5 × 5.0 × 4.0 cm | Surgery | Vimentin (+) | ND | (+) | Surgery, radiotherapy, and chemotherapy | Alive 42 months after surgery |
| Magishi et al. (19) | Female / 74 | Asymptomatic | Left upper lung, 5.7 × 5.0 × 3.3 cm | Surgery | ND | (−) | Performed after surgery | Surgery | Died 11 months after surgery |
| Kadowaki et al. (20) | Male / 72 | Dyspnea, chest pain | Left lower lung, 9.0 × 9.0 cm | Needle biopsy | Vimentin (+), AE1/AE3 (−), S-100 (−), CD56 (−), CD34 (−) | (+) | (+) | ND | Died 3 months after symptom onset |
|  | Male / 77 | Hemoptysis | Left lower lung, 11.0 × 8.0 cm | Needle biopsy | Vimentin (+), Osteocalcin (+), S-100 (focal+), AE1/AE3 (−) | (+) | (+) | ND | Died 3 months after symptom onset |
| Wei et al. (21) | Male / 65 | Cough, right-sided chest pain | Right upper lung, 12.0 × 10.0 × 6.0 cm | Surgery | Vimentin (+), CK (−) | ND | ND | Surgery | Alive 4 months after surgery |
| Yamazaki et al. (22) | Male / 78 | Cough, hemoptysis | Left upper lung, 7.2 × 7.0 × 6.2 cm | Surgery | Vimentin (+), Osteonectin (+), AE1/AE3 (−), EMA (−), Desmin (−), S-100 (−) | ND | (+) | Surgery | Died 7 months after surgery |
| Han et al. (23) | Female / 53 | Chest tightness, wheezing | Right upper lung, 5.0 × 5.0 × 4.0 cm | Surgery | Vimentin (+), CD99 (+), CK (−), Desmin (−), S-100 (−), Syn (−), CgA (−), LCA (−) | ND | ND | Surgery | ND |
| Shenjere et al. (24) | Female / 56 | Cough | Right lower lung, 3.2 × 2.2 × 2.0 cm | Surgery | Vimentin (+), CK7 (−), CK20 (−), EMA (−), CEA (−) | ND | ND | Surgery | ND |
|  | Female / 58 | Cough, chest pain | Left lower lung, 25.0 × 24.0 × 6.0 cm | Surgery | Vimentin (focal+), CK7 (−), CK5/6 (−), EMA (−), TTF-1 (−), S-100 (−), SMA (−) | ND | ND | Surgery | ND |
|  | Male / 66 | Detected during evaluation for nerve compression | Left upper lung, 6.0 × 5.0 cm | Biopsy | Vimentin (+++), CD99 (+), αSMA (focal+), S-100 (−), Desmin (−), CAM5.2 (−), AE1/AE3 (−) | ND | ND | Surgery | Alive 12 months after initial presentation |
|  | Male / 79 | Shortness of breath | Left upper lung, 11.0 × 9.0 cm | Biopsy | Vimentin (+++), CD99 (+), αSMA (focal+), S-100 (−), Desmin (−), CAM5.2 (−), AE1/AE3 (−) | ND | (+) | Radiotherapy | Died 5 months after initial presentation |
| Gu et al. (25) | Male / 58 | Chest pain | Right middle lobe, 5.7 × 8.0 × 5.3 cm | Surgery | ND | (+) | (+) | Surgery and chemotherapy | ND |
| Wang (26) | Male / 52 | Left-sided chest pain, hemoptysis | Left upper lung, 10 × 7.0 × 6.0 cm | Surgery | ND | (+) | ND | Surgery | Alive 8 months after surgery |
| Takamura et al. (27) | Male / 80 | ND | Right middle and lower lobes, 14 cm | Autopsy | Vimentin (+), PCK (−) | ND | ND | Radiotherapy and chemotherapy | Died |
| Qi and Sun (28) | Male / 77 | Cough | Left lower lung, 5.0 × 6.0 × 7.0 cm | Surgery | Vimentin (+), Ki-67 proliferation index approximately 20%-30% | ND | ND | Surgery | ND |
| Zhao and Zheng (29) | Male / 17 | Cough, hemoptysis | Left lower lung, 4.2 × 4.0 × 3.8 cm | Surgery | ND | (+) | (+) | Surgery and chemotherapy | Alive 5 years after surgery |
| Wen et al. (30) | Male / 17 | Cough, hemoptysis | Left lower lung, 4.2 × 4.0 × 3.8 cm | Surgery | Vimentin (3+), Ki-67 proliferation index approximately 40%, EMA (−), SMA (−), Desmin (−), TTF-1 (a few scattered cells+), S-100 (−), CD34 (−) | (+) | (+) | Surgery and chemotherapy | Alive 8 years after surgery |
| Yang et al. (31) | Male / 61 | ND | Right upper lung, 1.8 × 1.6 × 1.5 cm | Surgery | SATB2 (3+), Ki-67 proliferation index approximately 30%, p53 (diffuse strong+), PCK (−), Desmin (−), S100 (1+), SMA (1+), CD34 (endothelium only) | (−) | (+) | Surgery and chemotherapy | Alive 9 months after surgery |
| Current case | Male / 58 | Cough, dyspnea | Strip-like high-density shadow in the left main bronchus | Bronchoscopic biopsy | SATB2 (+), Vimentin (+), P16 (diffuse strong+), p53 (diffuse strong+), Ki-67 proliferation index approximately 30%, S-100 (−), SOX-10 (−), MyoD1 (−), MDM2 (−), CD34 (−), CD68 (−), SMA (−) | (−) | (+) | Radiotherapy | Alive 9 months after diagnosis |

ND, not documented

**References**

1．Greenspan EB. Primary osteoid chondrosarcoma of the lung report of a case. *Am J Cancer*. (1933) 18:603-9.
2．Yamashita T, Kiyota T, Ukishima G, Hayama T, Yabuki E. Autopsy case of chondroosteoidsarcoma originating in the lung. *Showa Igakkai Zasshi*. (1964) 23:472-3.
3．Nosanchuk JS, Weatherbee L. Primary osteogenic sarcoma in lung: report of a case. *J Thorac Cardiovasc Surg*. (1969) 58:242-7. doi: 10.1016/S0022-5223(19)42608-1
4．Reingold IM, Amromin GD. Extraosseous osteosarcoma of the lung. *Cancer*. (1971) 28:491-8. doi: 10.1002/1097-0142(197108)28:2<491::aid-cncr2820280231>3.0.co;2-7
5．Nascimento AG, Unni KK, Bernatz PE. Sarcomas of the lung. *Mayo Clin Proc*. (1982) 57:355-9.
6．Bagaric I, Belicza M. Extraskeletal osteogenic sarcoma of the lungs. *Lijec Vjesn*. (1982) 104:467-70.
7．Saito H, Sakai S, Kobayashi T, Ishioroshi Y, Uno Y. Primary osteosarcoma of the lung-a case report. *Nihon Kyobu Shikkan Gakkai Zasshi*. (1983) 21:153-6.
8．Zhu JG, Yan ZK, Su YY. [Primary pulmonary osteosarcoma: a case report]. *Chin J Oncol*. (1984) 6:18. doi: 10.3760/cma.j.issn.0253-3766.1984.01.124 (in Chinese)
9．Colby TV, Bilbao JE, Battifora H, Unni KK. Primary osteosarcoma of the lung. A reappraisal following immunohistologic study. *Arch Pathol Lab Med*. (1989) 113:1147-50.
10．Loose JH, el-Naggar AK, Ro JY, Huang WL, McMurtrey MJ, Ayala AG. Primary osteosarcoma of the lung. Report of two cases and review of the literature. *J Thorac Cardiovasc Surg*. (1990) 100:867-73. doi: 10.1016/S0022-5223(19)36829-1
11．Petersen M. Radionuclide detection of primary pulmonary osteogenic sarcoma: a case report and review of the literature. *J Nucl Med*. (1990) 31:1110-4.
12．Stark P, Smith DC, Watkins GE, Chun KE. Primary intrathoracic extraosseous osteogenic sarcoma: report of three cases. *Radiology*. (1990) 174:725-6. doi: 10.1148/radiology.174.3.2305056
13．Bhalla M, Thompson BG, Harley RA, McLoud TC. Primary extraosseous pulmonary osteogenic sarcoma: CT findings. *J Comput Assist Tomogr*. (1992) 16:974-6. doi: 10.1097/00004728-199211000-00027
14．Miller DL, Allen MS. Rare pulmonary neoplasms. *Mayo Clin Proc*. (1993) 68:492-8. doi: 10.1016/S0025-6196(12)60199-2
15．Wei HQ, Guo RH. [Primary pulmonary osteosarcoma: a case report]. *Chin J Oncol*. (1996) 18:44. doi: 10.3760/cma.j.issn.0253-3766.1996.01.128 (in Chinese)
16．Wagner MS, Reyes CV. Primary chondroblastic osteosarcoma of the lung. *Sarcoma*. (1999) 3:193-5. doi: 10.1080/13577149977640
17．Sievert LJ, Elwing TJ, Evans ML. Primary pulmonary osteogenic sarcoma. *Skeletal Radiol*. (2000) 29:283-5. doi: 10.1007/s002560050609
18．Chapman AD, Pritchard SC, Yap WW, Rooney PH, Cockburn JS, Hutcheon AW, et al. Primary pulmonary osteosarcoma: case report and molecular analysis. *Cancer*. (2001) 91:779-84. doi: 10.1002/1097-0142(20010215)91:4<779::AIDCNCR1064>3.0.CO;2-J
19．Magishi K, Yoshida H, Izumi Y, Ishikawa N, Kubota H. Primary osteosarcoma of the lung: report of a case. *Surg Today*. (2004) 34:150-2. doi: 10.1007/s00595-003-2651-y
20．Kadowaki T, Hamada H, Yokoyama A, Katayama H, Abe M, Nishimura K, et al. Two cases of primary pulmonary osteosarcoma. *Intern Med*. (2005) 44:632-7. doi: 10.2169/internalmedicine.44.632
21．Wei HS, Yue JH, Lu MS, Zeng SE, Yue CY. [Primary pulmonary osteosarcoma: a case report]. *J Diagn Pathol*. (2005) 12:49,93. (in Chinese)
22．Yamazaki K, Okabayashi K, Hamatake D, Maekawa S, Yoshida Y, Yoshino I, et al. Primary osteosarcoma of the lung: a case report. *Ann Thorac Cardiovasc Surg*. (2006) 12:126-8.
23．Han XJ, Li YH, Liu XY, Gong L, Zhu SJ, Yao L, et al. Pulmonary extraskeletal osteosarcoma: a case report and review of literature. *Mod Oncol*. (2010) 18:1326-8. (in Chinese)
24．Shenjere P, Travis WD, Franks TJ, Doran HM, Hasleton PS. Primary pulmonary osteosarcoma: a report of 4 cases and a review of the literature. *Int J Surg Pathol*. (2011) 19:225-9. doi: 10.1177/1066896909332382
25．Gu T, Shi H, Xiu Y, Gu Y. Primary pulmonary osteosarcoma: PET/CT and SPECT/CT findings. *Clin Nucl Med*. (2011) 36:e209-12. doi: 10.1097/RLU.0b013e3182291ec3
26．Wang DD. [Primary pulmonary osteosarcoma: a case report]. *Radiol Pract*. (2011) 26:467. doi: 10.13609/j.cnki.1000-0313.2011.04.040 (in Chinese)
27．Takamura K, Ogi T, Yamamoto M, Kikuchi K. Extraskeletal osteosarcoma of the lung following treatment of primary small-cell lung carcinoma with chemoradiotherapy: a case report. *Mol Clin Oncol*. (2018) 8:99-102. doi: 10.3892/mco.2017.1500
28．Qi ZY, Sun ZG. Primary pulmonary osteosarcoma: a case report. *Chin J Radiol*. (2020) 54:249-50. doi: 10.3760/cma.j.issn.1005-1201.2020.03.015 (in Chinese)
29．Zhao YT, Zheng R. Primary pulmonary osteosarcoma: a case report. *China Clin Case Database*. (2022) 4:E00765. doi: 10.3760/cma.j.cmcr.2022.e00765 (in Chinese)
30．Wen X, Xue L, Jiang X, Jiang J, Li M, Zhang L. Case report: a 17-year-old male with primary pulmonary osteosarcoma. *Front Med (Lausanne)*. (2024) 11:1364937. doi: 10.3389/fmed.2024.1364937
31．Yang X, Zhao WG, Gao C, Lin S. Primary pulmonary osteosarcoma presenting as a 1.7-cm subpleural nodule in a 61-year-old man: a case report. *Am J Case Rep*. (2025) 26:e949034. doi: 10.12659/AJCR.949034
